# Wavelet-Based Multiscale Intermittency Analysis: The Effect of Deformation

**DOI:** 10.3390/e25071080

**Published:** 2023-07-18

**Authors:** José M. Angulo, Ana E. Madrid

**Affiliations:** Department of Statistics and Operations Research, University of Granada, 18071 Granada, Spain; anaesther@ugr.es

**Keywords:** complexity, deformation, energy transfer, entropy, intermittency, wavelets

## Abstract

Intermittency represents a certain form of heterogeneous behavior that has interest in diverse fields of application, particularly regarding the characterization of system dynamics and for risk assessment. Given its intrinsic location-scale-dependent nature, wavelets constitute a useful functional tool for technical analysis of intermittency. Deformation of the support may induce complex structural changes in a signal. In this paper, we study the effect of deformation on intermittency. Specifically, we analyze the interscale transfer of energy and its implications on different wavelet-based intermittency indicators, depending on whether the signal corresponds to a ‘level’- or a ‘flow’-type physical magnitude. Further, we evaluate the effect of deformation on the interscale distribution of energy in terms of generalized entropy and complexity measures. For illustration, various contrasting scenarios are considered based on simulation, as well as two segments corresponding to different regimes in a real seismic series before and after a significant earthquake.

## 1. Introduction

Intermittency, generally understood as the pseudo-periodic occurrence of higher-level or variation episodes within a certain regular behavior, is considered a phenomenon of interest in very diverse fields of applications (e.g., seismology, turbulence, hydrology, astronomy, finance, insurance, epidemiology, etc.). Structural characteristics associated with such an effect, in relation to the underlying generating process, often constitute a primary objective in environmental studies, as they provide relevant information for the detection and prediction of critical events and for risk assessment.

Different manifestations of intermittency have lead to various interpretations and formal definitions of this concept. These are essentially related to its genesis and, as a consequence, to the nature of its effects as a certain form of heterogeneous behavior. Depending on the domain where such heterogeneities occur, it is common to distinguish between ‘isolated’ types of intermittency, for heterogeneities in the spatial/temporal domain, and ‘non-isolated’ types of intermittency, associated with heterogeneous scaling, closer to the concept of multifractality (see, for example, [1]). A common feature of these approaches is then the heterogeneity in the energy distribution of a signal over space/time and/or scales, noting that both effects can be present in a given signal. This fact justifies the use of wavelet functions and related tools to analyze intermittency. In particular, in the context of assessment of a temporal signal, we use the well-known location-scale-dependent intermittency indicator introduced in [2,3], named as the ‘local intermittecy measure’, as well as its scale-dependent temporal average, called the ‘flatness factor’ by Meneveau [4]. The latter is a special case of sparsity wavelet-moment-based measure of intermittency; see, for instance, [5,6,7]. Among other contributions of interest in this context, see, for example, [6,8,9,10,11,12,13] and references therein.

Deformation has been used, among other purposes, as an approach to define flexible classes of nonhomogeneous random fields from homogeneous ones (e.g., [14,15,16], etc.), or in image warping techniques as a means to approximate heterogeneously behaved processes in terms of simpler homogeneous models (e.g., [17,18], etc.). Different aspects of the effect of space and space–time deformation are analized, for instance, in [19,20,21,22,23].

Information measures play a key role in the assessment of structural complexity of a signal in diverse fields of application. Different formulations have followed after the seminal paper by Shannon [24], among which the generalizacions by Rényi [25], under preservation of extensivity, and Tsallis [26], in a non-extensive context, have had a special impact. In particular, information measures constitute the basis for construction of certain forms of complexity measures and interpretations under the notion of diversity [27]; see, for instance, Refs. [28,29] and references therein.

The main aim of this paper is to study the effect of time deformation on the structure of intermittecy in a given signal. Formally, deformation is assumed to be defined by a regular transformation of the time domain in terms of a diffeomorphism with a positive Jacobian. We distinguish the cases of ‘level’- and ‘flow’-type magnitudes. Specifically, we analyze local energy transfer between scales due to local contraction/dilation, and its subsequent implications in the structure of intermittency, particularly in relation to the above mentioned quantitative indicators. Furthermore, we assess the effect of deformation on the heterogeneous interscale distribution of energy based on Shannon, Rényi and Tsallis entropies, as well as in terms of generalized complexity measures.

The remainder of the paper is structured as follows. Section 2 introduces fundamental preliminary aspects, including wavelet-based quantifiers of intermittency, information measures and definitions of ‘level’- and ‘flow’-type deformation. In the Section 3, we analyze the interscale transfer of energy derived from the local contraction/dilation properties of deformation, and the effect on the intermittency indicators considered. Some illustrative examples are shown in Section 4, firstly considering simulated signals generated from an ARMA model structure with Gaussian or Cauchy input noise, and its second-order integrated ARIMA version, and secondly based on a real data seismic signal contrasting the results for two periods corresponding to different activity regimes. Finally, some conclusions and open directions for continuing research are commented on in Section 5.

## 2. Preliminaries

### 2.1. Intermittency Wavelet-Based Quantifiers

Wavelet-based techniques constitute a powerful tool for analyzing possible intermittency in a signal, and have been applied in many studies, in particular in the context of environmental sciences. An interesting related insight was given, for example, in [2,3]. Let x(t) be a signal with finite total energy,

$$E_{x}=\int_{-\infty }^{\infty }x{\left(t\right)}^{2}dt<\infty .$$

The continuous wavelet transform (CWT) is obtained through convolution between x(t) and all the possible versions, scaled and translated, of a basic wavelet function ψ. As interpreted by Meneveau [4], a wavelet transform coefficient W(a,b) can then be seen as a measure of the ‘correlation’ between the signal x(t) and ψ(t) at scale *a* and location *b*. The total energy of x(t) can be decomposed in terms of the wavelet coefficient energies, $E_{w}\left(\lambda \right)$, as

$$\begin{array}{cc} E_{x} & =\int_{0}^{\infty }E_{w}\left(\lambda \right)d\lambda =\int_{0}^{\infty }\left(\frac{1}{2\pi C_{\psi }}\int_{-\infty }^{\infty }{\left|W_{x}\left(a\left(\lambda \right),b\right)\right|}^{2}db\right)d\lambda \\ \; & =2\pi \int_{0}^{\infty }\frac{E_{w}\left(a\right)}{a^{2}}da=\frac{1}{C_{\psi }}\int_{0}^{\infty }\int_{-\infty }^{\infty }\frac{|W_{x}{\left(a,b\right)|}^{2}}{a^{2}}dbda, \end{array}$$

where λ=2π/a and $C_{\psi }=\int_{-\infty }^{\infty }\frac{{\left|\hat{\psi }(\lambda )\right|}^{2}}{\left|\lambda \right|}d\lambda <\infty$, with $\hat{\psi }\left(\lambda \right)=\int_{-\infty }^{\infty }\psi \left(t\right)e^{-i\lambda t}dt$. That is, a density for the scale-time distribution of the energy of the signal x(t), through scales *a* and locations *b*, is obtained from the square of $W_{x}\left(a,b\right)$, named ‘scalogram’. Taking into account that intermittency occurs when the energy is not evenly distributed, Farge (et al.) [2,3] introduced the so-called ‘local intermittency measure’ (LIM), defined by normalization of the scalogram with respect to the average local energy observed within each scale:

(1) $$LIM_{x}\left(a,b\right)=\frac{|W_{x}{\left(a,b\right)|}^{2}}{{\mathrm{Ave}}_{b}\left[|W_{x}{\left(a,b\right)|}^{2}\right]}.$$

If $LIM_{x}\left(a,b\right)=1$ for any *a* and *b*, then the energy of the function is equally distributed in scale and time, and the signal does not exhibit intermittent behavior. On the contrary, a coefficient $LIM_{x}\left(a,b\right)>1$ indicates that this given scale-location pair (a,b) contributes significantly, in relative terms, to the average. Therefore, the $LIM_{x}\left(a,b\right)$ map can be seen as the fundamental analysis of energy information, which can be further explored and processed to look for certain patterns or characteristics related to the concept of intermittency (interscale coherence, recurrence, persistence, event distribution patterns, etc.).

An immediate indicator of intermittency is given by the temporal average of $LIM_{x}^{2}\left(a,b\right)$, which defines the ‘flatness’ of the wavelet coefficients within each scale *a* (see [4]):

$$F_{x}\left(a\right)={\mathrm{Ave}}_{b}\left[LIM_{x}^{2}\left(a,b\right)\right]=\frac{{\mathrm{Ave}}_{b}\left[|W_{x}{\left(a,b\right)|}^{4}\right]}{{\left({\mathrm{Ave}}_{b}\left[|W_{x}{\left(a,b\right)|}^{2}\right]\right)}^{2}}.$$

The flatness $F_{x}$ is then a scale-dependent measure of the kurtosis of the input signal. A relatively high $F_{x}\left(a\right)$ value is taken as a possible indication of intermittency at scale *a*. Locally, the condition $LIM_{x}^{2}\left(a,b\right)>3$ can be used as a basic criterion to identify those events contributing to departure from Gaussianity in the distribution of wavelet coefficients. Nicolleau and Vassilicos [1] explain that often a signal is interpreted as intermittent when $F_{x}\left(a\right)$ increases for decreasing *a* since an intermittent signal displays activity over only a fraction of time, and this portion decreases with the scale *a* under consideration. A limitation of $F_{x}\left(\cdot \right)$ is that it does not discriminate intermittencies of isolated or non-isolated types.

It must be noted that the flatness factor is a special prominent case of general sparsity wavelet-moment-based measures of intermittency, defined by

(2) $$\mu_{x;p,q}\left(a\right)=\frac{{\mathrm{Ave}}_{b}\left[|W_{x}{\left(a,b\right)|}^{p}\right]}{{\left({\mathrm{Ave}}_{b}\left[|W_{x}{\left(a,b\right)|}^{q}\right]\right)}^{p/q}},\;p,q\geq 1,$$

with $F_{x}\left(a\right)$ corresponding to p=4, q=2 (see, for example, [5]).

As often mentioned in the literature, the CWT provides a complete description of a signal at all scales and locations, and it is then usually adopted when the user wants to depict the signal behavior at selected scale-space ranges. As is well-known, when an efficient non-redundant compression of information, yet allowing the signal reconstruction, is the objective, a particularly interesting alternative is given by the orthogonal discrete wavelet transform (DWT) (see, e.g., [30]).

In our analysis on the effect of deformation in terms of the scalogram and related intermittency indicators, we use the CWT to give a complete representation for a ‘continuous’ (in practice, for a finely discretized) scale-space range.

A key aspect in the analysis through wavelets is the choice of the mother wavelet, since the main objective of this type of analysis is to extract, by convolution, certain characteristics of interest in the structure of a signal. Therefore, the wavelet function should be selected depending on the target features to be detected. For example, it is known that the Haar wavelet is appropriate to identify a sustained change in the signal level, while the Morlet wavelet is more appropriate to seek concentration in time–frequency of signal energy (see [31]). This is a complementary issue that does not directly affect to the central lines and objectives of this study in relation to the effect of deformation on intermittency.

### 2.2. Information and Complexity Measures

For a discrete probability distribution $\bar{p}=\left(p_{1},\cdots ,p_{n}\right)$, Shannon entropy [24] is defined as

$$H\left(\bar{p}\right)=-\sum_{i=1}^{n}p_{i}\ln\left(p_{i}\right)=-E\left[\ln\left(\bar{p}\right)\right].$$

The minimum and maximum values of *H* are $H_{min}=0$ and $H_{max}=\ln\left(n\right)$, respectively, with $H_{min}$ being related to degenerate systems concentrating the probability mass in only one of the possible states, and $H_{max}$ being reached only in the case of equiprobability.

Two important generalizations of Shannon entropy, both based on a deformation parameter determining a power distortion on the argument distribution, are given by Rényi and Tsallis entropies.

Specifically, Rényi entropy [25] of order *q* of the discrete probability distribution $\bar{p}$ is defined as

$$H_{q}^{R}\left(\bar{p}\right)=\frac{1}{1-q}\ln\left(\sum_{i=1}^{n}p_{i}^{q}\right)=\frac{1}{1-q}\ln\left(E[{\bar{p}}^{q-1}]\right)\;\left(q\neq 1\right).$$

As before, the minimum and maximum values of $H_{q}^{R}$ are ${H_{q}^{R}}_{min}=0$ and ${H_{q}^{R}}_{max}=\ln\left(n\right)$.

On the other hand, Tsallis entropy [26] of order *q* of the discrete probability distribution $\bar{p}$ is defined as

$$H_{q}^{T}\left(\bar{p}\right)=\frac{1}{q-1}\left(1-\sum_{i=1}^{n}p_{i}^{q}\right)=\frac{1}{q-1}\left(1-E[{\bar{p}}^{q-1}]\right)\;\left(q\neq 1\right).$$

The minimum and maximum values of $H_{q}^{T}$ are ${H_{q}^{T}}_{min}=0$ and ${H_{q}^{T}}_{max}=\frac{1-n^{1-q}}{q-1}$.

Shannon entropy is the limiting case of Rényi and Tsallis entropies as q→1.

Whilst Rényi entropy is extensive, meaning that is additive for independent systems, Tsallis entropy is non-extensive except for q→1.

Based on Rényi entropy, a product-type generalized complexity measure can be defined as

(3) $$C_{\alpha ,\beta }\left(\bar{p}\right)=e^{H_{\alpha }^{R}\left(\bar{p}\right)-H_{\beta }^{R}\left(\bar{p}\right)},$$

for 0<α,β<∞ (see [32], in the continuous case; see also [29] and references therein).

In the context of this work, these measures provide useful tools for complementary assessment of the distribution of energy, both within each particular scale and between different scales. In Section 4, we show, in particular, the effect of deformation on the interscale distribution of energy obtained from the DWT, considering Shannon, Rényi and Tsallis entropies normalized by their corresponding maximum value (see, e.g., [11], for the case of normalized Tsallis wavelet entropy), as well as the above mentioned Rényi-entropy-based formulation of a product-type complexity measure.

### 2.3. Deformation

Let *x* be a signal on [0,T], and let Φ be a deformation of the domain [0,T], subject to the constraints that it is continuously differentiable with strictly positive derivative $\Phi^{\prime }$. This ensures that there is no time reversal, nor time ruptures, nor any local collapse of the Lebesgue measure on single points. For simplicity, we assume that $\Phi \left(\left[0,T\right]\right)=\left[0,T^{\prime }\right]$. We distinguish the cases where *x* represents either a ‘level’- or a ‘flow’-type magnitude, that is, depending on whether the states represent values that are intrinsic to specific time points or are accumulated during increasing time periods. In correspondence, we denote x[Φ] and $x[\tilde{\Phi }]$ the transformed signals defined on $\left[0,T^{\prime }\right]$, respectively defined by

$$x\left[\Phi \right]\left(t^{\prime }\right)=x\left(\Phi^{-1}\left(t^{\prime }\right)\right)$$

and

$$x\left[\tilde{\Phi }\right]\left(t^{\prime }\right)=x\left(\Phi^{-1}\left(t^{\prime }\right)\right){\left[\Phi^{\prime }\left(\Phi^{-1}\left(t^{\prime }\right)\right)\right]}^{-1},$$

with $t^{\prime }\in \left[0,T^{\prime }\right]$. Whilst in x[Φ] the signal is only affected by displacement of the time points, in $x[\tilde{\Phi }]$ the contraction/dilation properties of Φ affect the accumulation process and hence are reflected in the modification of the state values through the local first derivative, with the latter being smaller than 1 under local contraction, and larger than 1 in the case of local dilation.

In relation to the formal definition of a transformed random signal by deformation of a *d*-dimensional space support, with a distinction between the cases of ‘level’- and ‘flow’-type magnitudes, see, for instance, [21,22,23].

## 3. Deformation and Intermittency: Interscale Transfer of Energy

In order to explain induced heterogeneity and intermittency changes derived from deformation, we first specifically analyze interscale transfer of energy effects in relation to local contraction/dilation properties of such transformation. For this purpose, we consider a simple linear transformation $\Phi :\left[0,T\right]\to \left[0,T^{\prime }\right]$, defined by Φ(t)=ct, with c>0 a real constant, and $T^{\prime }=cT$. Hence, Φ(t) is a contraction for c<1 and a dilation for c>1.

We distinguish the cases of deformation on a ‘level’- or a ‘flow’-type signal, according to the formulations given in Section 2.3.

Recall that wavelet coefficients are obtained by the convolution of the signal with a given rescaled and translated wavelet function, that is,

$$W_{x}\left(a,b\right)=\langle x\left(t\right),\psi_{ab}\left(t\right)\rangle =\frac{1}{a^{1/2}}\int_{0}^{T}x\left(t\right)\psi \left(\frac{t-b}{a}\right)dt.$$

Then, the wavelet coefficients of the deformed signal x[Φ] (‘level’ case) are obtained substituting x[Φ](u) for x(t) in the previous expression:

(4) $$\begin{array}{cc} W_{x[\Phi ]}\left(a,b\right)\; & \;=\;\langle x\left[\Phi \right]\left(u\right),\psi_{ab}\left(u\right)\rangle =\frac{1}{a^{1/2}}\int_{0}^{cT}x\left[\Phi \right]\left(u\right)\psi \left(\frac{u-b}{a}\right)du\\ \; & \;=\;\frac{1}{a^{1/2}}\;\int_{0}^{cT}\;x\;\left(\;\frac{u}{c}\;\right)\;\psi \;\left(\;\frac{u-b}{a}\;\right)\;du\;=\;\frac{1}{a^{1/2}}\;\int_{0}^{T}\;x\left(t\right)\psi \;\left(\;\frac{ct-b}{a}\;\right)\;cdt\\ \; & \;=\;\frac{c}{a^{1/2}}\;\int_{0}^{T}\;x\;\left(t\right)\psi \;\left(\;\frac{t\;-\;b/c}{a/c}\;\right)\;dt\;=\;\frac{c^{1/2}}{\;\left(\;a/c\right){\;}^{1/2}}\;\int_{0}^{T}\;x\;\left(t\right)\psi \;\left(\;\frac{t\;-\;b/c}{a/c}\;\right)\;dt\\ \; & =c^{1/2}W_{x}\left(\frac{a}{c},\frac{b}{c}\right), \end{array}$$

where we have used the change of variable u/c=t in the fourth equality. This relation between the wavelet coefficients of the original and deformed signals indicates that, without considering the change of domain, there is a transfer of energy from scale *a* to scale ca. In fact, averaging the squared wavelet coefficients over *b*, using the change of variable $b^{\prime }=\frac{b}{c}$, and then renaming $b^{\prime }\to b$, we get

(5) $$\begin{array}{cc} {\mathrm{Ave}}_{b}\left[W_{x[\Phi ]}^{2}\left(a,b\right)\right] & =\frac{1}{cT}\int_{0}^{cT}W_{x[\Phi ]}^{2}\left(a,b\right)db=\frac{1}{cT}\int_{0}^{cT}{\left(c^{1/2}\right)}^{2}W_{x}^{2}\left(\frac{a}{c},\frac{b}{c}\right)db\\ \; & =\frac{1}{T}\int_{0}^{T}W_{x}^{2}\left(\frac{a}{c},b^{\prime }\right)cdb^{\prime }=\frac{c}{T}\int_{0}^{T}W_{x}^{2}\left(\frac{a}{c},b\right)db\\ \; & =c{\mathrm{Ave}}_{b}\left[W_{x}^{2}\left(\frac{a}{c},b\right)\right]. \end{array}$$

Substituting the expressions (4) and (5) in (1), the LIM of the deformed signal is computed as

(6) $$LIM_{x[\Phi ]}\left(a,b\right)\;=\;\frac{W_{x[\Phi ]}^{2}\left(\;a,b\;\right)}{{\mathrm{Ave}}_{b}\;\left[\;W_{x[\Phi ]}^{2}\left(\;a,b\;\right)\;\right]}\;=\;\frac{cW_{x}^{2}\;\left(\;\frac{a}{c},\frac{b}{c}\;\right)\;}{c{\mathrm{Ave}}_{b}\;\left[\;W_{x}^{2}\left(\frac{a}{c},b\;\right)\right]}\;=\;LIM_{x}\;\left(\;\frac{a}{c},\frac{b}{c}\;\right).$$

Hence, we can assert that if the deformation has a local effect of dilation (c>1), then the relative energy value of scale a/c at location b/c is transferred to the higher scale a>a/c at location *b*. On the other hand, if the deformation has a local effect of contraction (c<1), there is a transfer of energy from scale a/c at location b/c to the lower scale a<a/c at location *b*. Note that, due to the change of measure between the original and the transformed physical spaces, the total energy is expanded or shortened by deformation depending on the properties of dilation or contraction, as it can be seen using the changes of variables $a^{\prime }=\frac{a}{c}$ and $b^{\prime }=\frac{b}{c}$:

(7) $$\begin{array}{cc} E_{x[\Phi ]} & \;=\;\frac{1}{C_{\psi }}\;\int_{0}^{\infty }\;\;\int_{-\infty }^{\infty }\;\frac{|W_{x[\Phi ]}{\left(a,\;b\right)|}^{2}}{a^{2}}dadb\;=\;\frac{1}{C_{\psi }}\;\int_{0}^{\infty }\;\;\int_{-\infty }^{\infty }\;\frac{c|W_{x}\left(\frac{a}{c},\;\frac{b}{c}\right){\;|}^{2}}{a^{2}}dadb\\ \; & =\;\frac{c}{C_{\psi }}\;\;\int_{0}^{\infty }\;\int_{-\infty }^{\infty }\;\frac{|W_{x}\left(a^{\prime },\;b^{\prime }\right){|}^{2}}{{\left(ca^{\prime }\right)}^{2}}cda^{\prime }cdb^{\prime }\;=\;\frac{c}{C_{\psi }}\;\int_{0}^{\infty }\;\;\int_{-\infty }^{\infty }\;\frac{|W_{x}\left(a^{\prime },\;b^{\prime }\right){|}^{2}}{a^{\prime 2}}da^{\prime }db^{\prime }\\ \; & =cE_{x}. \end{array}$$

For a general deformation Φ, depending on where we have local contraction or dilation effects, there will be transfers of energy between scales in different directions.

A similar analysis can be developed for *x* representing a ‘flow’-type signal. In this case, we obtain the following expressions for $x[\tilde{\Phi }]$:

$$W_{x[\tilde{\Phi }]}\left(a,b\right)=\langle x\left[\tilde{\Phi }\right]\left(u\right),\psi_{ab}\left(u\right)\rangle =\frac{1}{c}W_{x[\Phi ]}\left(a,b\right)=\frac{1}{c^{1/2}}W_{x}\left(\frac{a}{c},\frac{b}{c}\right),$$

$${\mathrm{Ave}}_{b}\left[W_{x[\tilde{\Phi }]}^{2}\left(a,b\right)\right]={\mathrm{Ave}}_{b}\left[\frac{1}{c^{2}}W_{x[\Phi ]}^{2}\left(a,b\right)\right]=\frac{1}{c}{\mathrm{Ave}}_{b}\left[W_{x}^{2}\left(\frac{a}{c},b\right)\right],$$

(8) $$\begin{array}{c} LIM_{x[\tilde{\Phi }]}\;\left(a,b\right)\;=\;\frac{|W_{x[\tilde{\Phi }]}{\;\left(a,b\right)\;|}^{2}}{{\mathrm{Ave}}_{b}\;\left[\;|W_{x[\tilde{\Phi }]}{\left(a,b\right)|}^{2}\;\right]\;}\;=\;\frac{\frac{1}{c}W_{x}^{2}\;\left(\frac{a}{c},\frac{b}{c}\right)\;}{\frac{1}{c}{\mathrm{Ave}}_{b}\;\left[\;W_{x}^{2}\;\left(\frac{a}{c},b\right)\;\right]\;}\;=\;LIM_{x}\;\left(\frac{a}{c},\frac{b}{c}\right). \end{array}$$

Comparing (6) and (8), we can see that the LIM values coincide in both cases. However, as is shown below, using the same changes of variables as before, the total energy change now is

$$\begin{array}{cc} E_{x[\tilde{\Phi }]} & =\frac{1}{C_{\psi }}\int_{0}^{\infty }\int_{0}^{cT}\frac{|W_{x[\tilde{\Phi }]}{\left(a,b\right)|}^{2}}{a^{2}}dadb=\frac{1}{C_{\psi }}\int_{0}^{\infty }\int_{0}^{cT}\frac{|W_{x}\left(\frac{a}{c},\frac{b}{c}\right){|}^{2}}{ca^{2}}dadb\\ \; & =\;\frac{1}{C_{\psi }}\int_{0}^{\infty }\;\int_{0}^{T}\;\frac{|W_{x}\left(a^{\prime },b^{\prime }\right){|}^{2}}{c{\left(ca^{\prime }\right)}^{2}}cda^{\prime }cdb^{\prime }\;=\;\frac{1}{C_{\psi }}\int_{0}^{\infty }\;\int_{0}^{T}\;\frac{|W_{x}\left(a^{\prime },b^{\prime }\right){|}^{2}}{ca^{\prime 2}}da^{\prime }db^{\prime }\;=\;\frac{E_{x}}{c}. \end{array}$$

Hence, when the signal is of ‘flow’-type, the energy is reduced where the deformation has local dilation properties, c>1, and enhanced where the deformation has local contraction properties, c<1. Note that in the case of a ‘level’-type signal, just the opposite effect was proved (see (7)).

As introduced in Section 2.1, a global multiscale quantification of intermittency is given in terms of the flatness coefficient *F*, which averages squared values of the local intermittency measures (LIM) obtained within each single scale. In the case of a ‘level’-type signal *x*, and for a deformation Φ(t)=ct as before, using the previous results, we have

$$\begin{array}{cc} F_{x[\Phi ]}\left(a\right) & ={\mathrm{Ave}}_{b}\left[LIM_{x[\Phi ]}^{2}\left(a,b\right)\right]=\frac{1}{cT}\int_{0}^{cT}LIM_{x[\Phi ]}^{2}\left(a,b\right)db\\ \; & =\frac{1}{cT}\int_{0}^{cT}LIM_{x}^{2}\left(\frac{a}{c},\frac{b}{c}\right)db=\frac{1}{cT}\int_{0}^{T}LIM_{x}^{2}\left(\frac{a}{c},b^{\prime }\right)cdb^{\prime }\\ \; & =\frac{1}{T}\int_{0}^{T}LIM_{x}^{2}\left(\frac{a}{c},b\right)db=F_{x}\left(\frac{a}{c}\right). \end{array}$$

This shows that the shape of the flatness curve is preserved, but on different scales.

For a ‘flow’-type signal *x*, since the LIM values coincided under both contraction or dilation, therefore the flatness coefficient values coincide too,

$$F_{x[\tilde{\Phi }]}\left(a\right)={\mathrm{Ave}}_{b}\left[LIM_{x[\tilde{\Phi }]}^{2}\left(a,b\right)\right]={\mathrm{Ave}}_{b}\left[LIM_{x[\Phi ]}^{2}\left(a,b\right)\right]=F_{x}\left(\frac{a}{c}\right).$$

Similar results can be obtained, under analogous developments, for the general sparsity-wavelet-moment-based measures of intermittency (2).

For a general non-linear deformation, with varying contraction or dilation effects, the local transfer of energy between scales leads, by the ${\mathrm{Ave}}_{b}$ operation involved, to different values of LIM and flatness coefficients depending on whether the signal is assumed to be of ‘level’- or ‘flow’-type, as is shown in Section 4.

## 4. Illustrative Cases

In this section, we study the multiscale effect of deformation on intermittency, first based on the analysis of several selected simulated scenarios. Secondly, two segments corresponding to different activity regimes in a real seismic signal are also analyzed and compared with this purpose.

### 4.1. Simulated Examples

In this subsection, we first consider two versions of the ARMA(1,1) model

(9) $$X_{t}=\phi_{1}X_{t-1}+\epsilon_{t}-\theta_{1}\epsilon_{t-1},\;\phi_{1}=-0.7,\;\;\theta_{1}=0.9,$$

respectively driven by Cauchy and Gaussian white noise $\left\{\epsilon_{t}\right\}$. Due to the negative first-order auto-regression parameter, as well as the alternating influence of driving noise inputs over time, the structure of the model induces fluctuations associated with prevalence of higher order frequencies in the power spectrum. On the other hand, it is expected that the random input energy supplied to the system in the heavy-tail Cauchy white noise case, in contrast with the Gaussian white noise case, leads to the presence of clustered high-variability episodes in the generated realizations.

**Figure 1:**
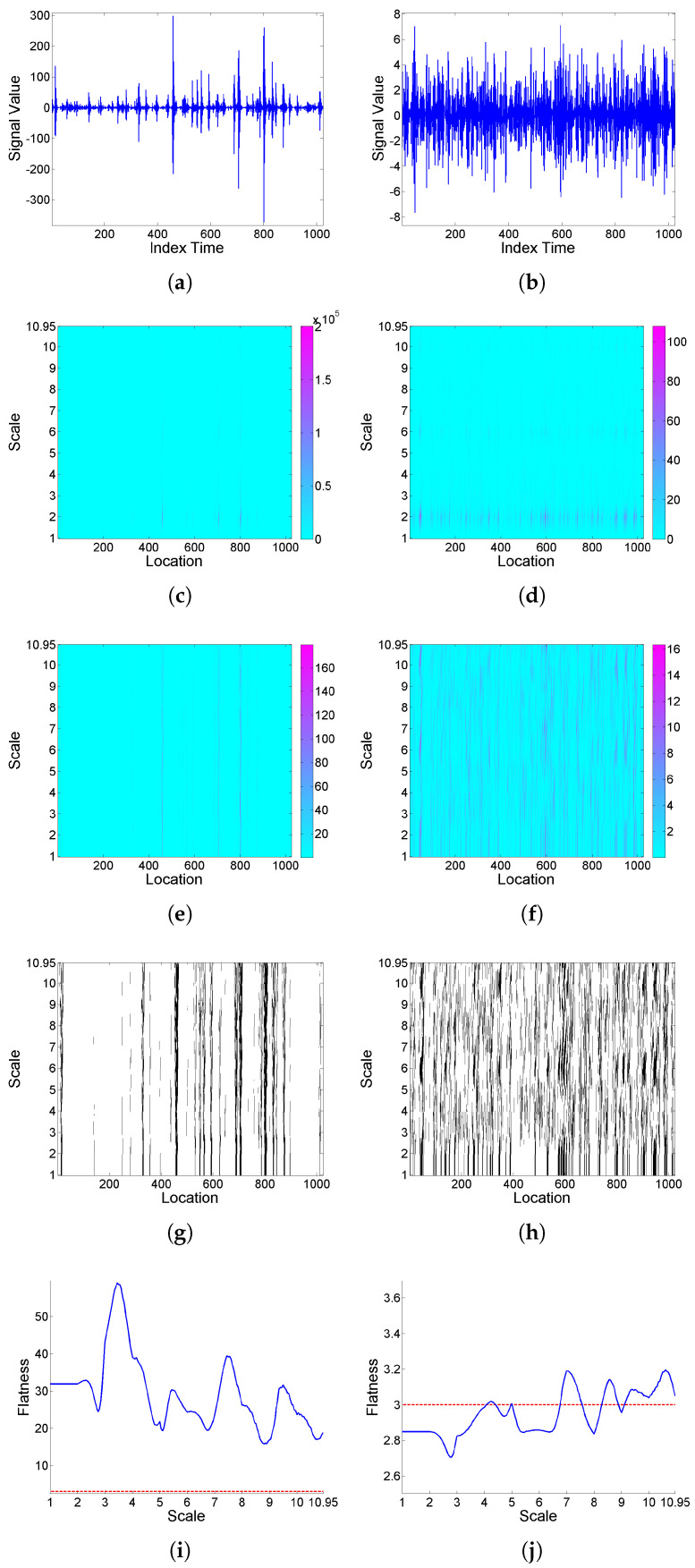
From top to bottom: (**a**,**b**) simulated realizations of model (9), with (**a**) Cauchy and (**b**) Gaussian white noise; correspondingly in left and right columns, using Haar wavelet, (**c**,**d**) scalogram $W_{x}^{2}\left(a,b\right)$, (**e**,**f**) $LIM_{x}$ map, (**g**,**h**) threshold exceedance set for $LIM_{x}^{2}\left(a,b\right)>3$, and (**i**,**j**) $F_{x}$ curve.

Figure 1 illustrates, for two simulated realizations of model (9) (left and right plots respectively correspond to the Cauchy and Gaussian white noise cases), the analysis of intermittency using the quantifiers introduced in Section 2.1 based on a Haar wavelet. Specifically, from top to bottom, the simulated realizations, corresponding scalograms, LIM maps, threshold exceedance set for $LIM_{x}^{2}\left(a,b\right)>3$, and *F* curves are plotted. In the case where the noise is Cauchy a well-defined interscale coherence of energy concentrations over time can be observed, and a strong global intermittency behavior is clear from the *F* curve. However, when the noise is Gaussian, the behavior is more regular, the LIM values are similar through scales and locations, indicating that the energy of the function is homogeneously distributed in time within each scale, and the *F* curve shows slightly significant values only for some higher scales.

**Figure 2:**
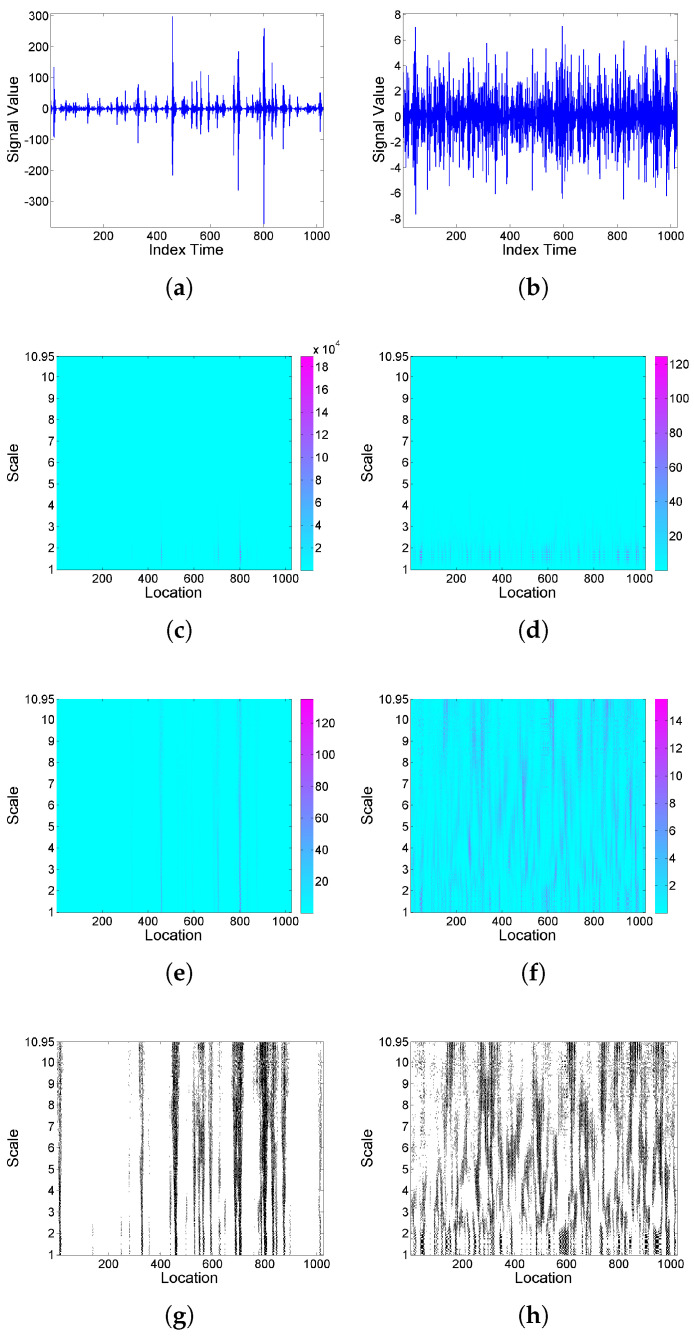

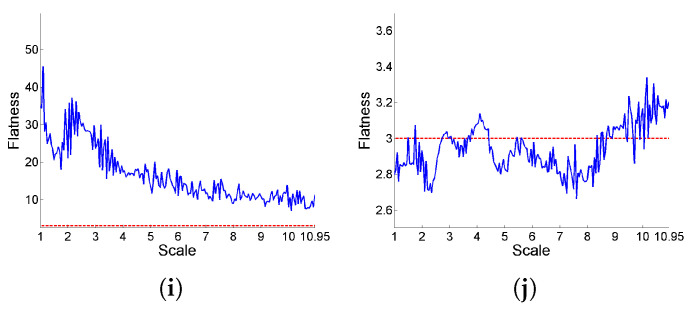
From top to bottom: (**a**,**b**) simulated realizations of model (9), with (**a**) Cauchy and (**b**) Gaussian white noise; correspondingly in left and right columns, using Morlet wavelet, (**c**,**d**) scalogram $W_{x}^{2}\left(a,b\right)$, (**e**,**f**) $LIM_{x}$ map, (**g**,**h**) threshold exceedance set for $LIM_{x}^{2}\left(a,b\right)>3$, and (**i**,**j**) $F_{x}$ curve.

As commented at the end of Section 2.1, the wavelet function should be selected depending on the features to be detected. Figure 2 shows the results of a similar analysis to Figure 1, now using Morlet wavelet. In this case, the threshold exceedances for $LIM_{x}^{2}$ show a somewhat different structure with respect to the previous case, indicating less frequent concentrations of energy, but during longer time periods, and the *F* curves are much less smooth. In what follows, for illustration purposes, all the analyses are only based on a Haar wavelet.

To study the effect of deformation of the time domain (both ‘level’ and ‘flow’ cases are evaluated) on the distribution of the energy concentrations over time at different scales, we consider that the signals to be analyzed are observed at times t=1,2,…,1024, and, for simplicity, we apply a smooth deformation with increasing contraction and dilation effects respectively towards the left and right ends of the continuous time interval [0,1024], but preserving the domain. Formally, Φ is defined by its inverse, as

(10) $$\Phi^{-1}\left(t^{\prime }\right)=\frac{0.7\times 1024}{\pi }\sin\left(\frac{\pi }{1024}t^{\prime }\right)+t^{\prime },\;\mathrm{for}\;t\in \left[0,1024\right]$$

(see Figure 3).

**Figure 4:**
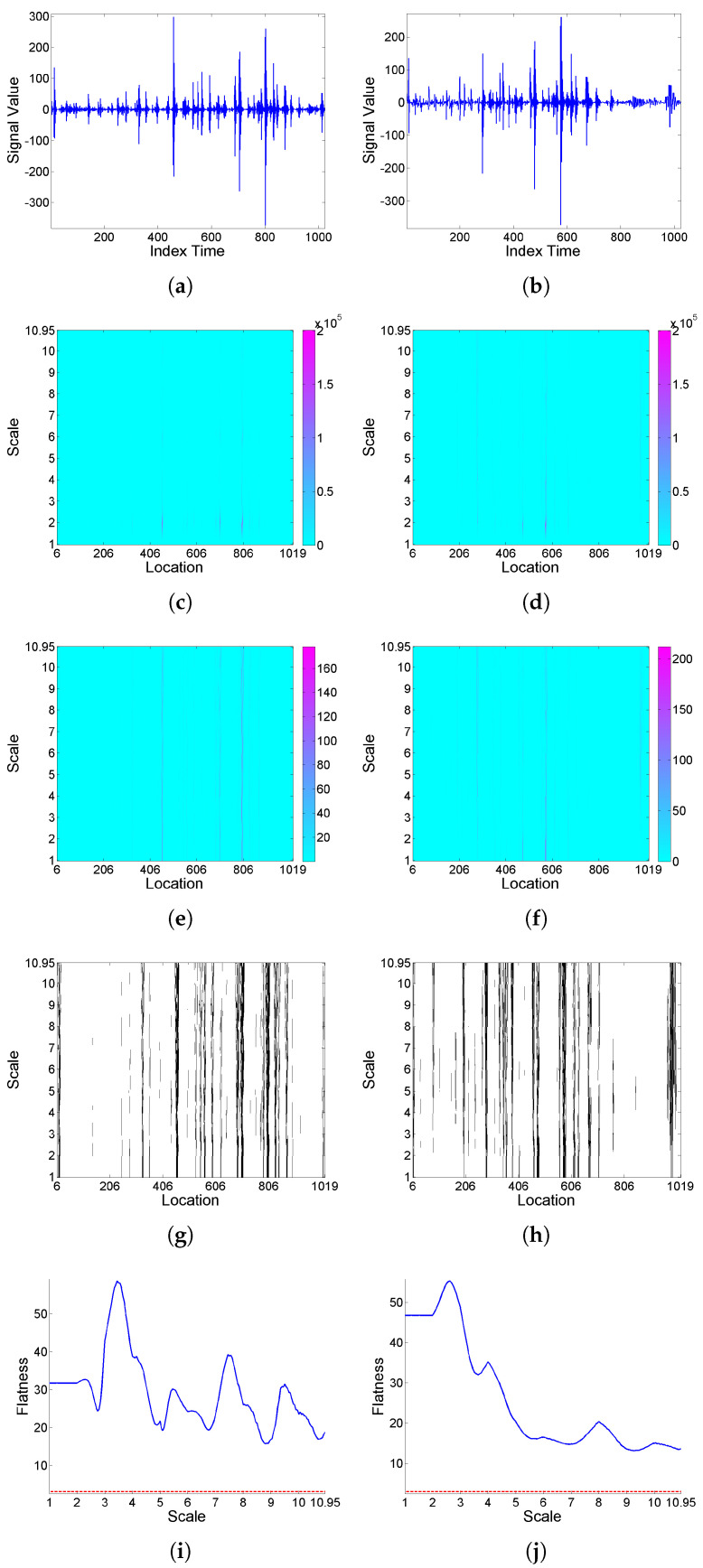
From top to bottom: (**a**) simulated signal realization *x* generated from model (9) with Cauchy white noise, and (**b**) its (‘level’-type) deformation x[Φ]; correspondingly in left and right columns, (**c**,**d**) scalogram $W^{2}\left(a,b\right)$, (**e**,**f**) LIM map, (**g**,**h**) threshold exceedance set for $LIM^{2}\left(a,b\right)>3$, and (**i**,**j**) *F* curve.

**Figure 5:**
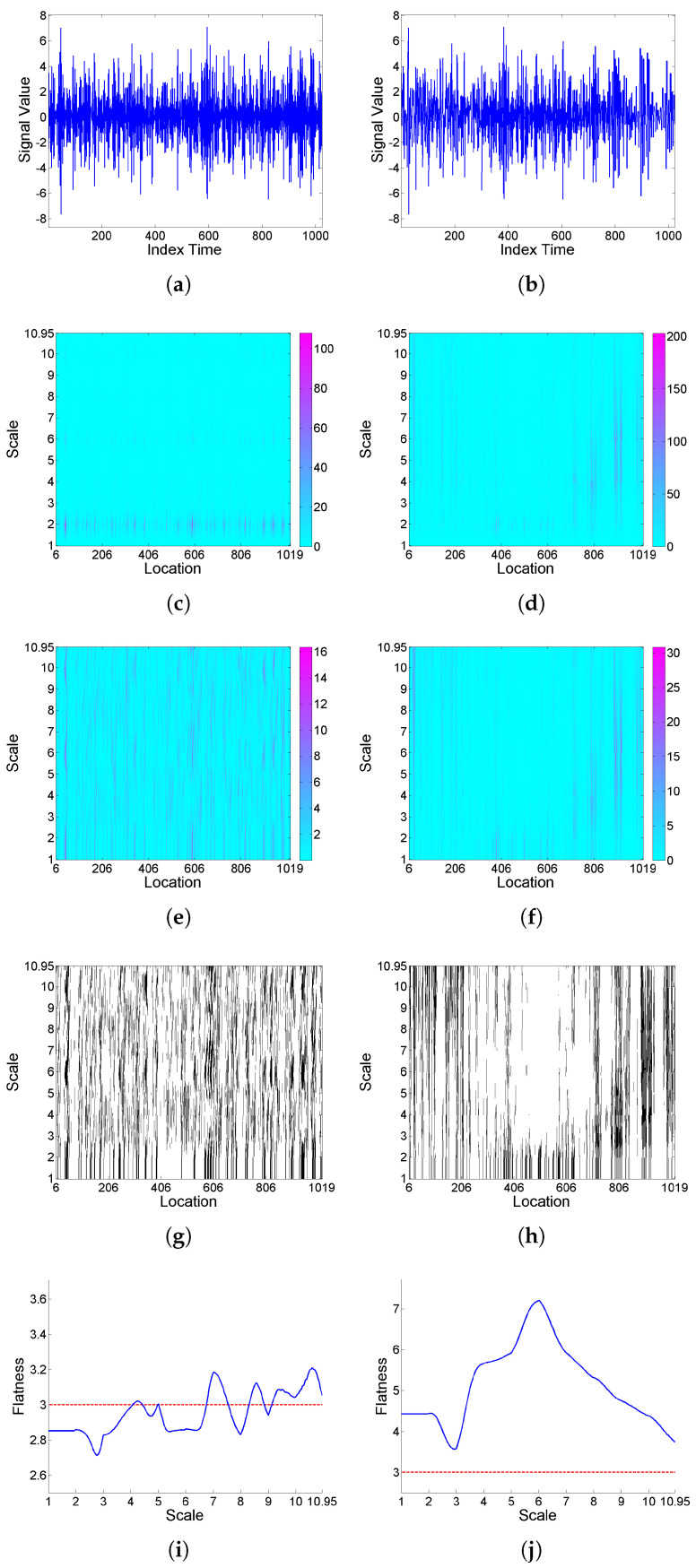
From top to bottom: (**a**) simulated signal realization *x* generated from model (9) with Gaussian white noise, and (**b**) its (‘level’-type) deformation x[Φ]; correspondingly in left and right columns, (**c**,**d**) scalogram $W^{2}\left(a,b\right)$, (**e**,**f**) LIM map, (**g**,**h**) threshold exceedance set for $LIM^{2}\left(a,b\right)>3$, and (**i**,**j**) *F* curve.

Firstly, the effect of deformation on a ‘level’-type signal is analyzed based on simulated realizations from ARMA(1,1) model (9) with Cauchy or Gaussian white noise; see Figure 4 and Figure 5, respectively. Looking at the *F* curves corresponding to the original signal and its transformation after deformation for the series with Cauchy noise, it can be seen that intermittency levels are modified under deformation. A similar effect is observed for the series with Gaussian noise, though in this case, with the original signal showing slightly significant values only for some higher scales, its transformation displays intermittency effects for all scales.

**Figure 6:**
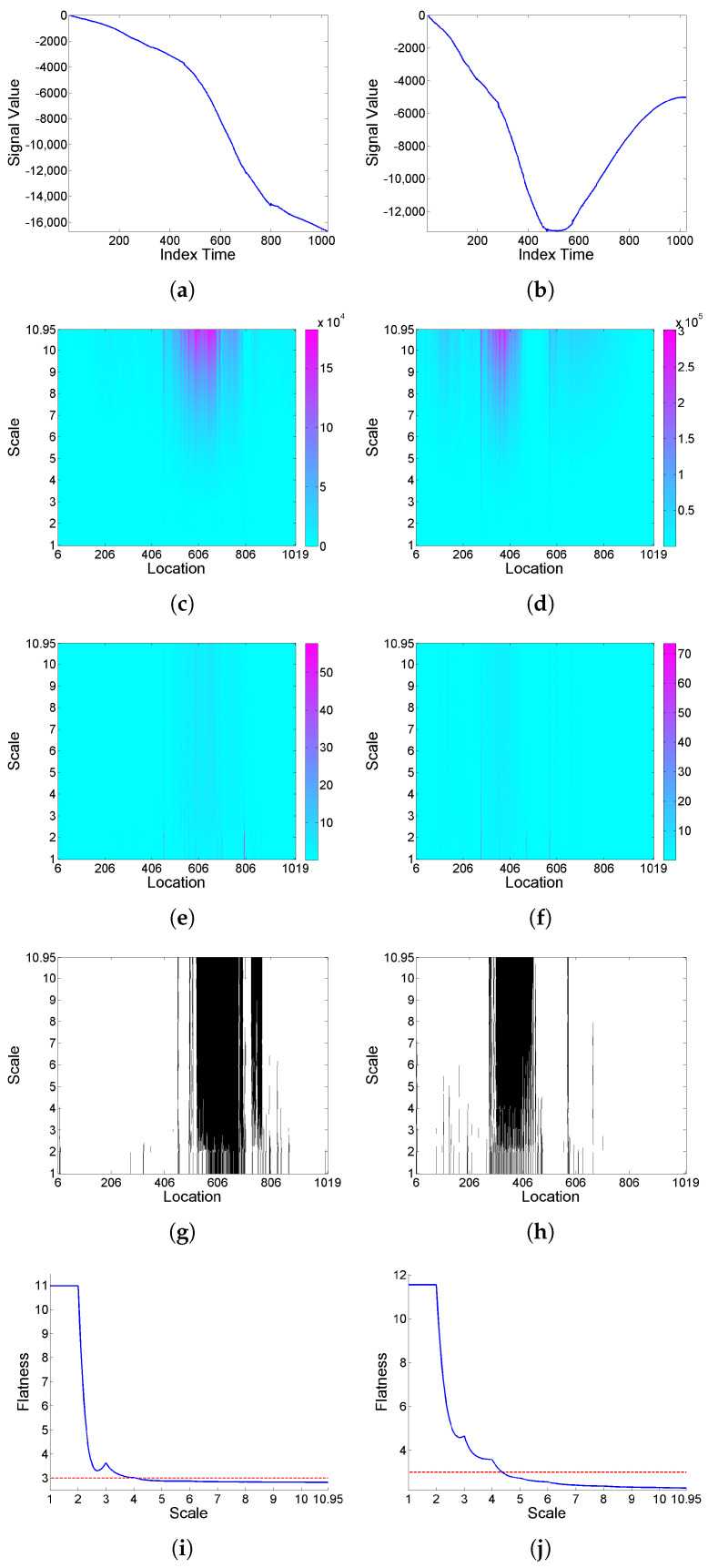
From top to bottom: (**a**) simulated signal realization *x* generated from the ARIMA(1,2,1) model obtained by double integration of model (9) with Cauchy noise, and (**b**) its (‘flow’-type) deformation $x[\tilde{\Phi }]$; correspondingly in left and right columns, (**c**,**d**) scalogram $W^{2}\left(a,b\right)$, (**e**,**f**) LIM map, (**g**,**h**) threshold exceedance set for $LIM^{2}\left(a,b\right)>3$, and (**i**,**j**) *F* curve.

**Figure 7:**
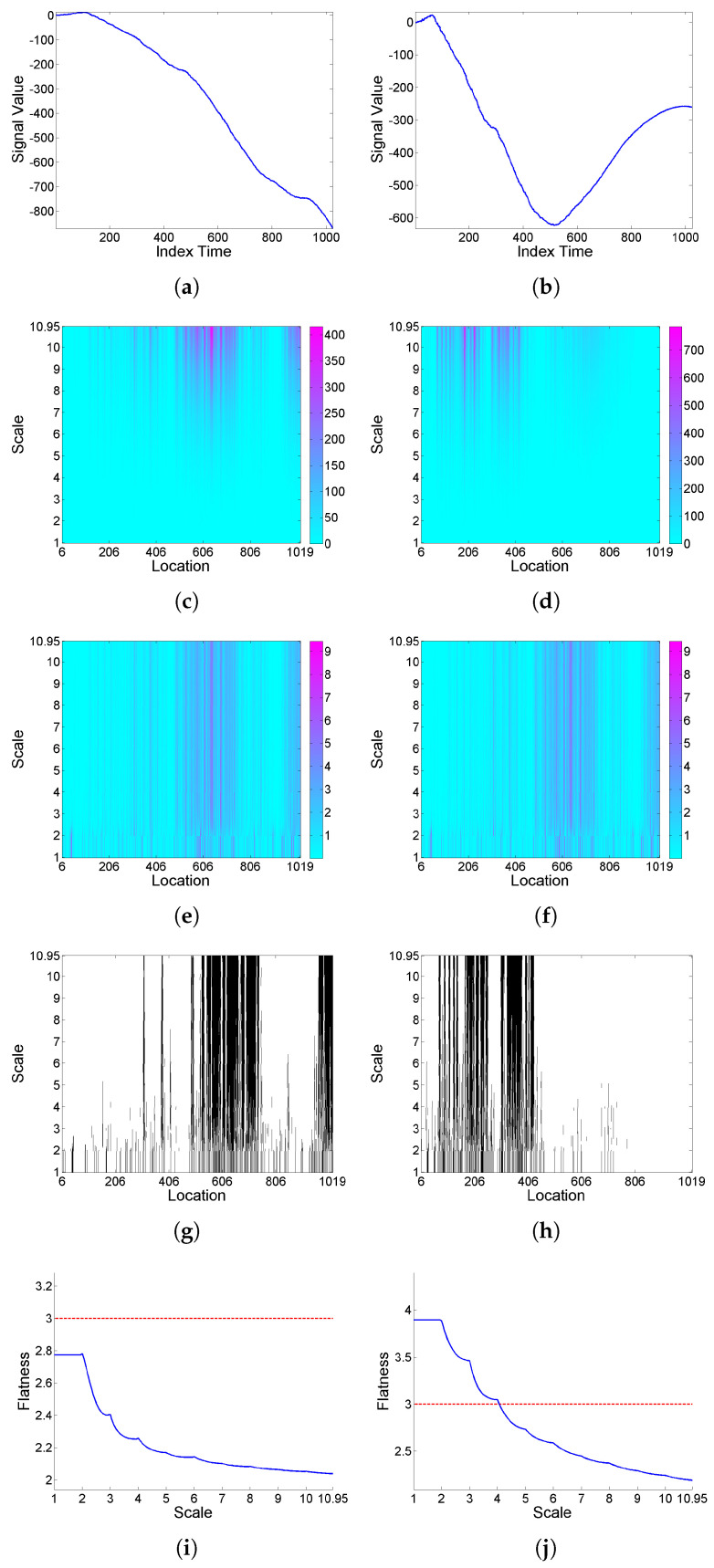
From top to bottom: (**a**) simulated signal realization *x* generated from the ARIMA(1,2,1) model obtained by double integration of model (9) with Gaussian noise, and (**b**) its (‘flow’-type) deformation $x[\tilde{\Phi }]$; correspondingly in left and right columns, (**c**,**d**) scalogram $W^{2}\left(a,b\right)$, (**e**,**f**) LIM map, (**g**,**h**) threshold exceedance set for $LIM^{2}\left(a,b\right)>3$, and (**i**,**j**) *F* curve.

Secondly, we consider series generated from the ARIMA(1,2,1) model obtained by double integration of ARMA model (9), again with Cauchy and Gaussian white noise, and study the effect of a ‘flow’-type deformation. In all cases, the *F* curves have a global decay for the range of scales considered. Also, a clear increase of intermittency levels is observed after deformation, as can be seen in Figure 6 and Figure 7, and particularly in the Gaussian noise case, which does not exhibit intermittency behavior originally.

Finally, a complementary analysis for intermittency is performed focusing on the assessment of temporal heterogeneities in the scale distribution of energy. For this purpose, as mentioned in Section 2.2, normalized wavelet entropy (Shannon, Rényi, Tsallis) is used to measure the degree of local structuring of a signal, based on sliding windows of size 128, with sliding step equal to 1. That is, for each time interval, entropy is calculated based on the distribution of relative energy values on the different scales, hence providing an indicator of possible structural changes over time.

**Figure 8:**
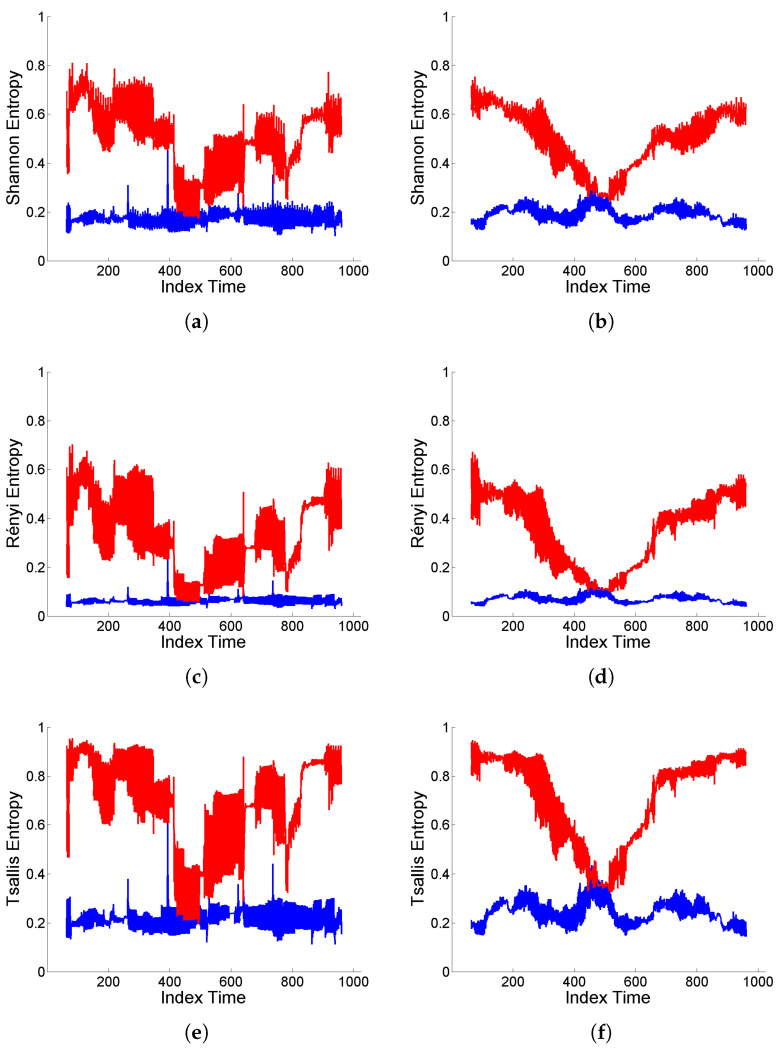
(**a**,**b**) Shannon entropy, (**c**,**d**) Rényi entropy of order q=3, and (**e**,**f**) Tsallis entropy of order q=3, displayed in blue color for original signal generated from model (9) with (**a**,**c**,**e**) Cauchy and (**b**,**d**,**f**) Gaussian white noise, and in red color for the corresponding (‘level’-type) deformation.

Figure 8 shows the wavelet entropy values based on the original and deformed ‘level’-type signals displayed in Figure 4 and Figure 5. It can be observed that, in both cases (Cauchy and Gaussian white noise), there is a high degree of structuring reflected in low values of wavelet entropies for the original signals, while either contraction or dilation effects of deformation induce an increase in entropy values (similar in shape, but different in level, for Shannon, Rényi and Tsallis entropies), indicating a higher homogeneity of the interscale energy distribution.

However, there are clear differences between both scenarios corresponding to Cauchy or Gaussian white noise. For the original signals, the entropy values are locally more sensitive to the sliding steps, although showing a more stable average level, in the Cauchy case. Furthermore, deformation enhances the intermittency effects of extremal inputs from the Cauchy white noise through the ARMA model, noticeable in the heterogeneous structure of the entropy curves.

**Figure 9:**
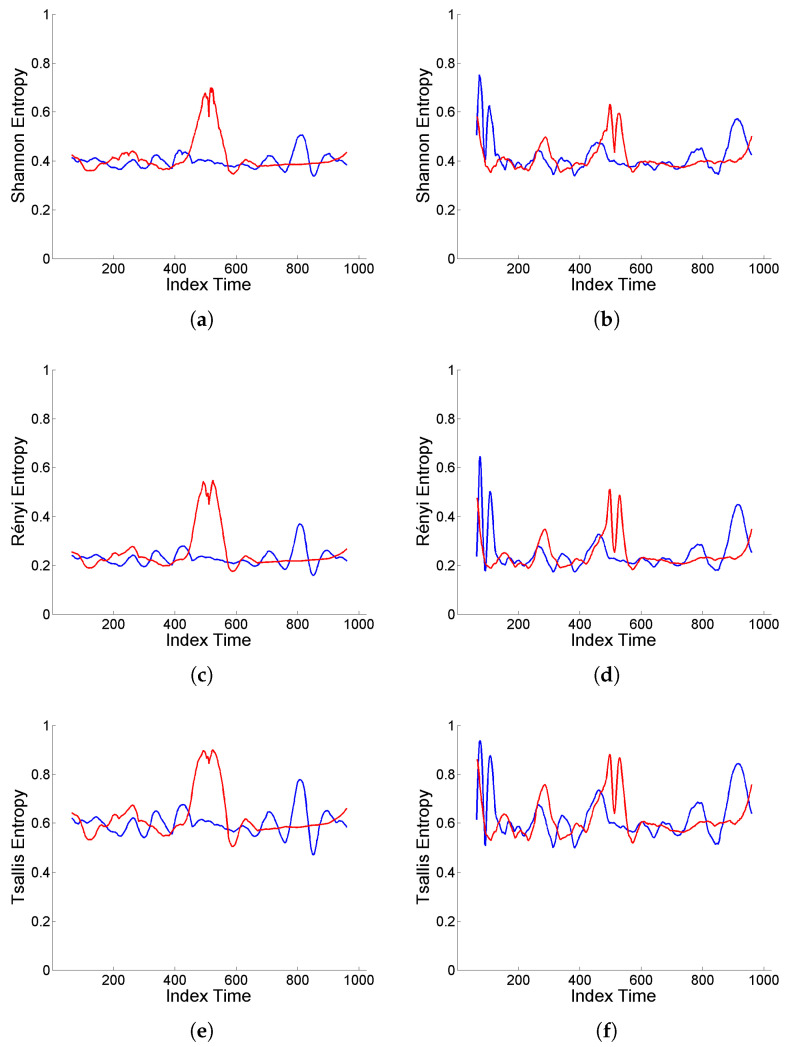
(**a**,**b**) Shannon entropy, (**c**,**d**) Rényi entropy of order q=3, and (**e**,**f**) Tsallis entropy of order q=3, displayed in blue color for original signal generated from the ARIMA(1,2,1) model obtained by double integration of model (9) with (**a**,**c**,**e**) Cauchy and (**b**,**d**,**f**) Gaussian white noise, and in red color for the corresponding (‘flow’-type) deformation.

An analogous analysis is performed under the ARIMA(1,2,1) model, based on the realizations displayed in top plots of Figure 6 and Figure 7, respectively, for the Cauchy and Gaussian white noise cases. Figure 9 shows the corresponding values of Shannon, Rényi and Tsallis entropies, which reflect a significantly smoother local behavior according to the nature of the signals. In these cases, the entropy values remain at a similar level for the original and deformed signals, except for the period where mixed contraction and dilation effects from deformation are present within the window.

### 4.2. Analysis of a Real Seismic Signal

**Figure 10:**
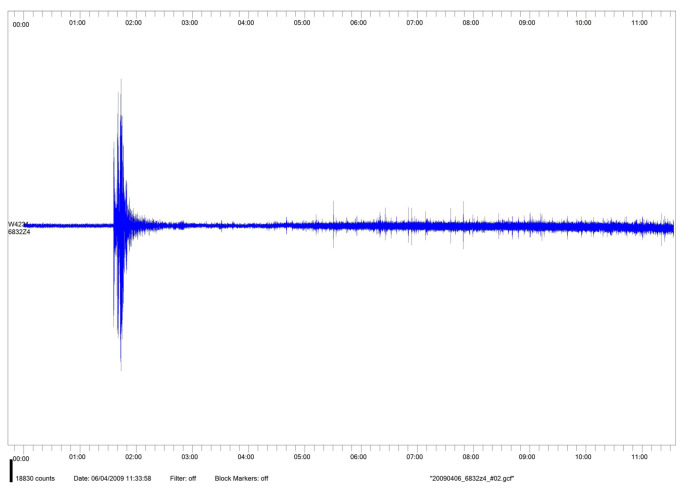
Seismic signal of L’Aquila earthquake (6 April 2009).

In this subsection, as an example with the aim of illustrating the interest of the aspects discussed above in a real situation, we consider the seismic signal (as it was registered in Granada, Spain) comprising the earthquake that happened on 6 April 2009, with epicenter near L’Aquila (Italy), with magnitude 6.3 on the Ritcher scale (see Figure 10).

**Figure 11:**
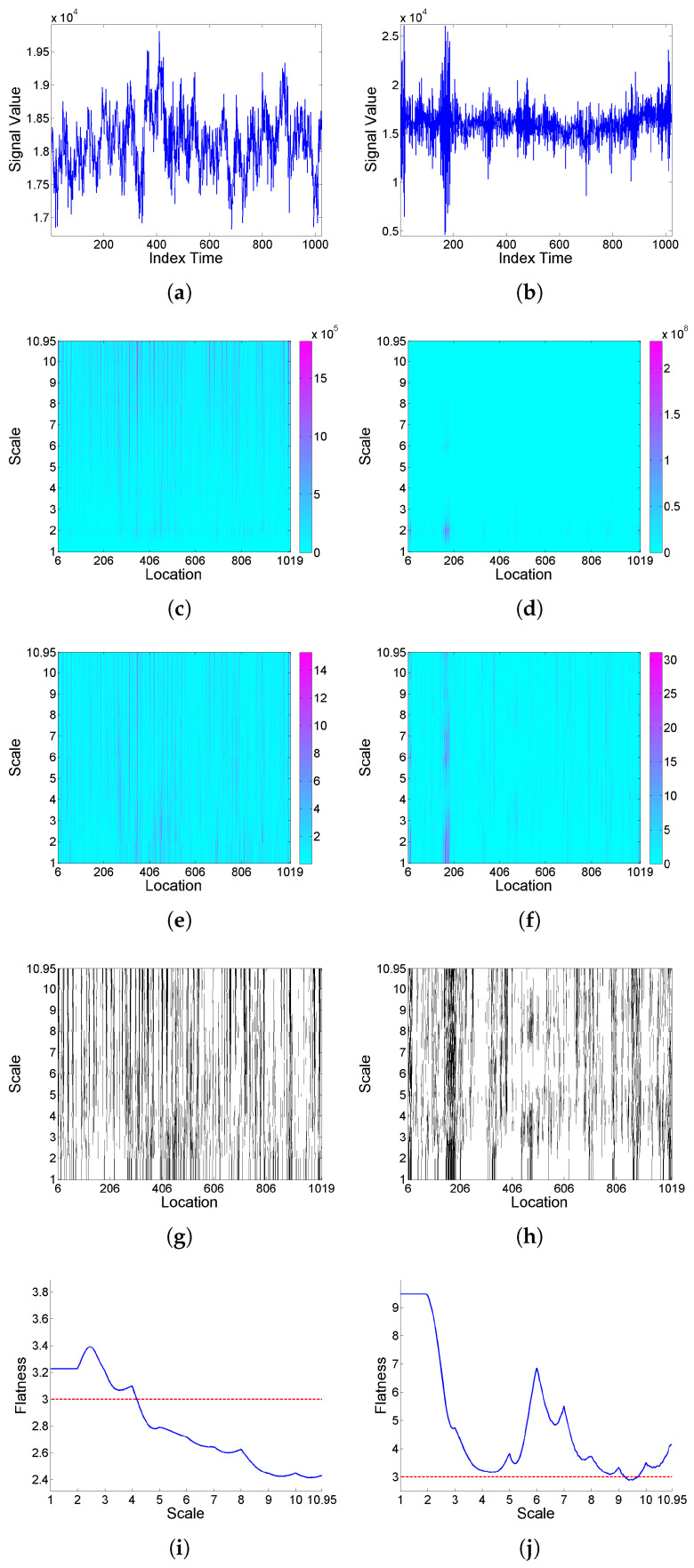
From top to bottom: (**a**) segment 1, (**b**) segment 2 of seismic signal; correspondingly in left and right columns, (**c**,**d**) scalogram $W_{x}^{2}\left(a,b\right)$, (**e**,**f**) $LIM_{x}$ map, (**g**,**h**) threshold exceedance set for $LIM_{x}^{2}\left(a,b\right)>3$, and (**i**,**j**) $F_{x}$ curve.

Since it is expected that the dynamics of the system differ before and after the earthquake, we select two segments for comparative analysis, respectively, beginning at 01:29:00 (segment 1, before) and 11:24:03 (segment 2, after). Figure 11 illustrates the analysis of intermittency, in terms of the scalogram, $LIM_{x}$ map, and $F_{x}$ curve. It can be observed that these indicators display different structural characteristics for both subperiods. In particular, the $LIM_{x}^{2}\left(a,b\right)$ excesses over threshold 3 depict clear local concentrations, with a certain degree of interscale coherency, in the aftershock segment. This is globally reflected in the much larger values of the flatness factor for this subperiod, which indicates a higher degree of intermittency after the earthquake.

Given the intrinsic nature of the signal, a ‘level’-type deformation according to Equation (10) is applied.

**Figure 12:**
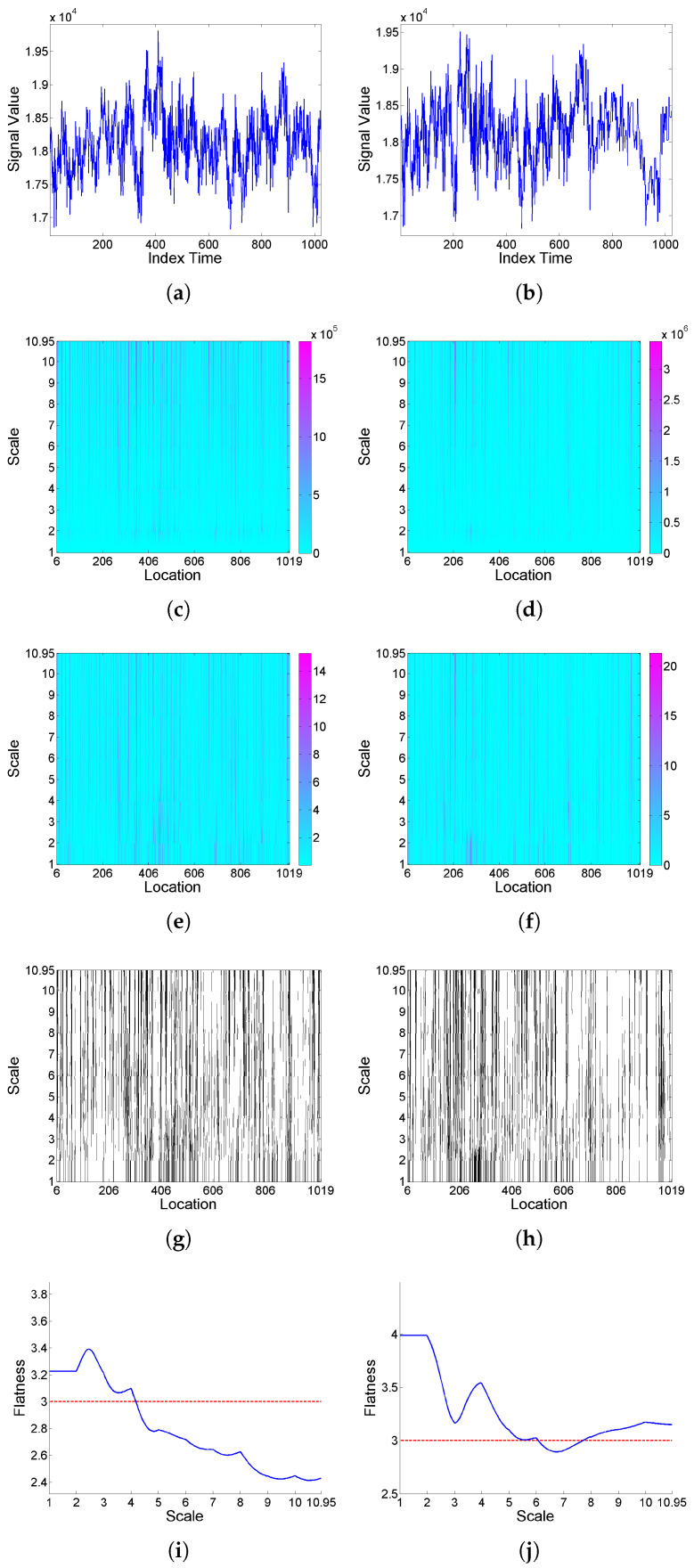
From top to bottom: (**a**) segment 1 of seismic signal, and (**b**) its (‘level’-type) deformation; correspondingly in left and right columns, (**c**,**d**) scalogram $W_{x}^{2}\left(a,b\right)$, (**e**,**f**) $LIM_{x}$ map, (**g**,**h**) threshold exceedance set for $LIM_{x}^{2}\left(a,b\right)>3$, and (**i**,**j**) $F_{x}$ curve.

**Figure 13:**
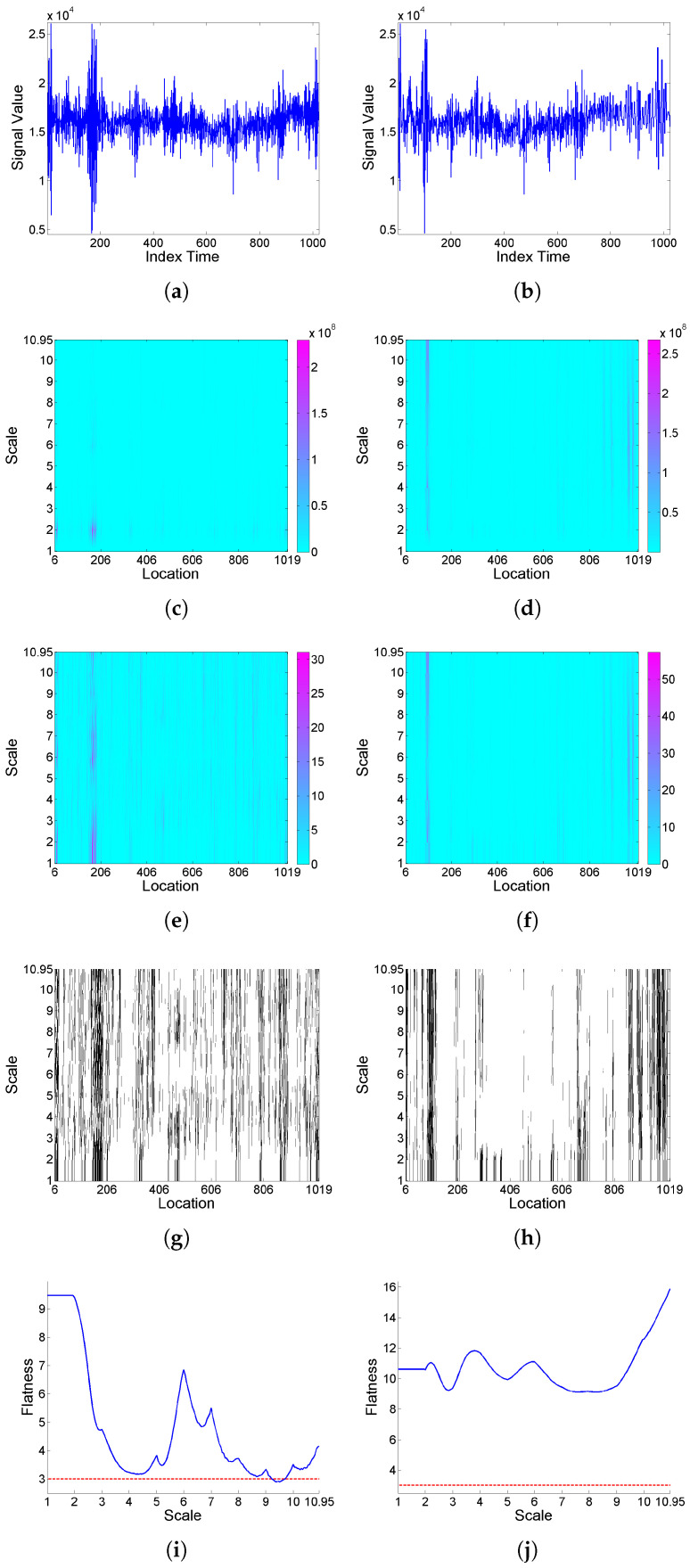
From top to bottom: (**a**) segment 2 of seismic signal, and (**b**) its (‘level’-type) deformation; correspondingly in left and right columns, (**c**,**d**) scalogram $W_{x}^{2}\left(a,b\right)$, (**e**,**f**) $LIM_{x}$ map, (**g**,**h**) threshold exceedance set for $LIM_{x}^{2}\left(a,b\right)>3$, and (**i**,**j**) $F_{x}$ curve.

The structural differences between both periods are maintained when the deformation is applied, as can be observed in Figure 12 and Figure 13. In general terms, the values of the *F* curves increase after deformation. While this effect is moderated for segment 1, yet with flatness values becoming larger than threshold 3 for most scales, there is a drastic increase for segment 2, particularly enhanced at lower scales.

**Figure 14:**
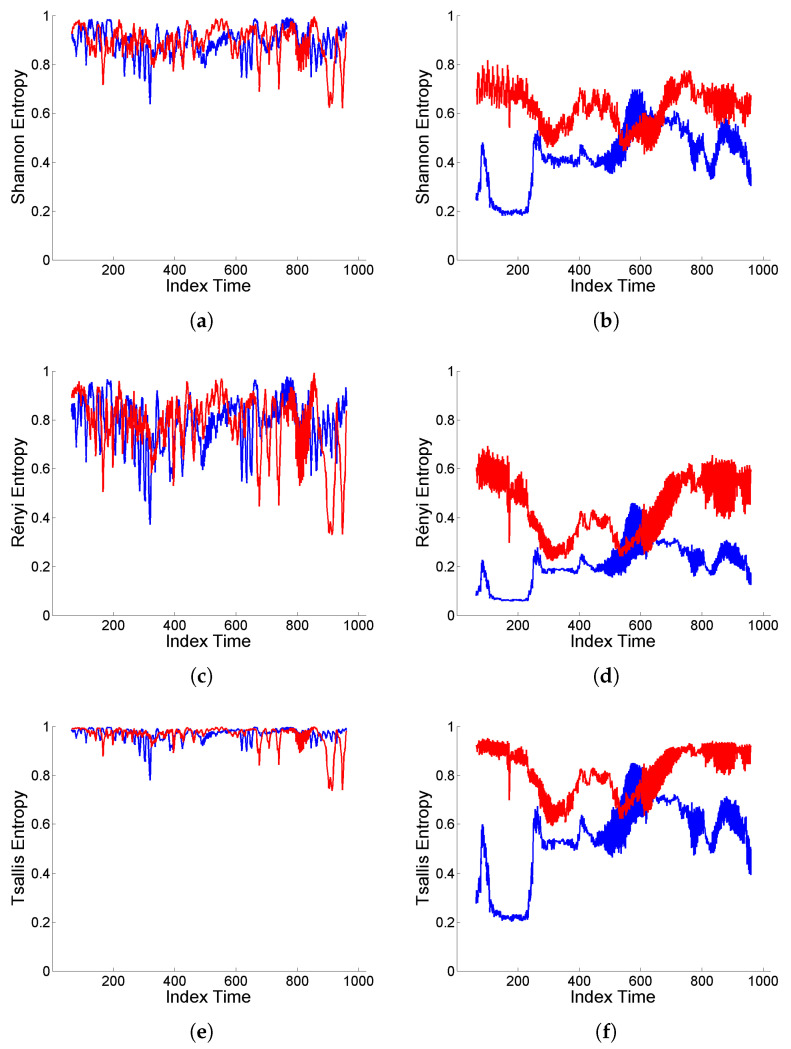
(**a**,**b**) Shannon entropy, (**c**,**d**) Rényi entropy of order q=3, and (**e**,**f**) Tsallis entropy of order q=3, displayed in blue color for original seismic signal with (**a**,**c**,**e**) segment 1 and (**b**,**d**,**f**) segment 2, and in red color for the corresponding (‘level’-type) deformation.

**Figure 15:**
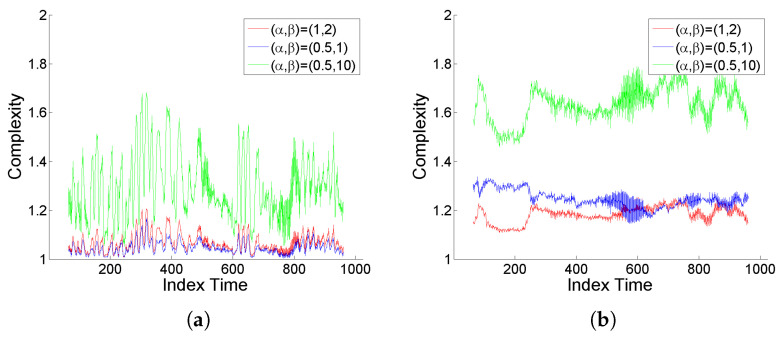

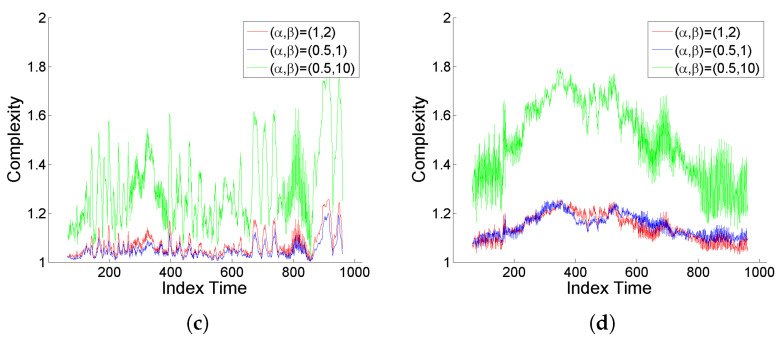
Rényi-based generalized complexity under selected (α,β) values, for (**a**,**b**) original seismic signal *x*, (**a**) segment 1 and (**b**) segment 2, and (**c**,**d**) for the corresponding (‘level’-type) deformation x[Φ].

Regarding the wavelet entropy analysis of the interscale relative energy distribution, it is interesting to note, as can be seen in Figure 14, that both periods, before and after the main seismic shock, show a very different behavior in agreement with the degree of structuring of the signal. For segment 1, the normalized entropy values are closer to the maximum, denoting a certain uniformity of the energy distribution, slightly affected by deformation. On the other hand, for segment 2, the entropy values are much lower, displaying temporal variations, and in this case deformation has a clear effect similarly as discussed for the simulated signals analyzed in Section 4.1. A clear distinction between both periods in relation to the intrinsic interscale energy distribution structure, as well as regarding the effect of deformation, can be also observed in Figure 15 based on the generalized complexity measure (3).

## 5. Conclusions

In this paper, we study the effect of time deformation on the structure of signals displaying an intermittent behavior. Regarding the nature of the signal, we distinguish the cases of ‘level’- and ‘flow’-type magnitudes. Specifically, under a wavelet-based approach, we analyze the interscale transfer of energy derived from local contraction or dilation properties of deformation, and its implications on well-known intermittency quantifiers such as the ‘local intermittency measure’ (LIM) and ‘flatness factor’ (F). Furthermore, variations in the heterogeneous interscale distribution of energy are assessed using Shannon, Rényi and Tsallis entropies, as well as the Rényi-entropy-based product-type generalized complexity measure.

In relation to the underlying generating random process, for an illustration based on simulation, we consider different scenarios regarding the dynamics dependence structure (ARMA, for a ‘level’-type magnitude, and its ARIMA second-order integrated version, for a ‘flow’-type magnitude), and the random inputs marginal distribution (Gaussian and Cauchy). In addition, as an example based on real data, we select two segments from a seismic series, respectively, before and after a big earthquake, hence corresponding to contrasting systemic activity regimes, and compare the effect of deformation on their intermittency behavior.

We must emphasise that our methodological approach is essentially empirical, in the sense that the wavelet-based analysis and extraction of information by different indicators is performed on observed or simulated signal data, which can be seen as realizations of a random system. Although we do not address any inferential objective in this paper, it is implicit that a researcher can potentially use this analytical approach under such perspective; for instance, in the case of repeated observations, or under model-based conditional simulation.

We can also identify, as a result of this work, some significant directions on which research can be continued. Among them, here we mention: dual determination of scale-dependent deformation functions representing the intermittency characteristics of a given signal; consideration of stochastic deformation (in particular, from covariate effects); sensitivity with respect to the deformation parameter *q* in generalized entropy measures (Rényi, Tsallis), and its potential usefulness for detecting intermittency levels from the inter/intrascale distribution of energy; extension and interpretation of results in terms of diversity and considering alternative complexity measures; intermittency analysis of spatial or spatiotemporal signals; risk analysis, e.g., using quantile-based risk measures (see, for example, [33]), on loss functions defined in terms of intermittency indicators, with projection, for instance, to identification of recurrence, persistency or clustering patterns; derivations under the perspective of multifractal analysis (see, for example, [34,35,36]).

## Figures and Tables

**Figure 3:**
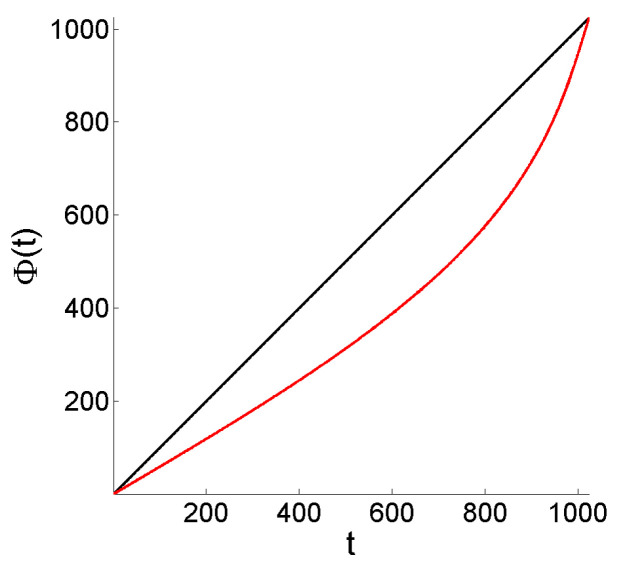
Deformation Φ given by (10).

## Data Availability

Seismic data used for analysis in Section 4.2 are available as supplementary material.

## Supplementary Materials

The following supporting information can be downloaded at: https://www.mdpi.com/article/10.3390/e25071080/s1.
